# The Ratio of Platelets to Lymphocytes Predicts the Prognosis of Metastatic Colorectal Cancer: A Review and Meta-Analysis

**DOI:** 10.1155/2021/9699499

**Published:** 2021-11-02

**Authors:** Jinming Wang, Jing Li, Sheng Wei, Jie Xu, Xiaohui Jiang, Lei Yang

**Affiliations:** ^1^Cancer Research Center Nantong, Nantong Tumor Hospital & Affiliated Tumor Hospital of Nantong University, Nantong, China; ^2^Department of General Surgery, Nantong Tumor Hospital & Affiliated Tumor Hospital of Nantong University, Nantong, China; ^3^Department of Oncology, Nantong Tumor Hospital & Affiliated Tumor Hospital of Nantong University, Nantong, China

## Abstract

**Background:**

In recent years, the incidence of colorectal cancer (CRC) has continued to increase. Although the overall prognosis of CRC has improved with the continuous improvement of the level of treatment, the prognosis of metastatic colorectal cancer (mCRC) is still poor. The purpose of our study is to explore the prognostic value of platelet to lymphocyte ratio (PLR) in mCRC.

**Methods:**

The PubMed, Web of Science, and Embase (via OVID) were systematically searched to obtain all relevant research. We used hazard ratio (HR) with 95% confidence interval (CI) to assess the associations of PLR and overall survival (OS) and progression free survival (PFS).

**Results:**

A total of twelve studies containing 1452 patients were included in this meta-analysis. Pooled analysis showed that high levels of PLR were associated with poor OS (HR: 1.72, 95% CI: 1.27–2.33, and *P* < 0.01) and PFS (HR: 1.64, 95% CI: 1.16–2.31, and *P* = 0.033).

**Conclusion:**

Our analysis suggested that high levels of PLR pretreatment may be an effective predictive biomarker for the prognosis of mCRC patients.

## 1. Introduction

Colorectal cancer (CRC) is one of the most common malignant tumors in the world. The latest global cancer statistics in 2020 show that the incidence of CRC ranks third among malignant tumors, and the mortality rate ranks second [1, 2]. China accounted for 24% of newly diagnosed cancer patients and 30% of global cancer-related deaths in 2020 [3]. Despite the increasing popularity of cancer screening and the improvement of diagnostic technology, 20% of CRC patients have already developed distant metastasis at the time of diagnosis [4]. The prognosis of patients with metastatic colorectal cancer (mCRC) is still poor, and the median overall survival (OS) is about 30 months [5]. Appropriate prognostic markers are needed to predict the prognosis of patients.

In recent years, more and more studies have proved that inflammation plays an important role in different stages of tumor development, including tumor occurrence, progression, malignant transformation, invasion, and metastasis [6–10]. Peripheral blood before diagnosis or treatment can reflect the inflammation of the tumor to a certain extent. It has been proved that platelets, lymphocytes, C-reactive protein, and Glasgow prognostic score as indicators of inflammation and immune response play a prognostic role in different tumors [11, 12]. As one of the inflammatory indicators, platelet to lymphocyte ratio (PLR) also has a certain predictive effect on the prognosis of tumors, such as pancreatic cancer, lung cancer, liver cancer, ovarian cancer, and breast cancer ([13–16]; X. [17]; Y. [18]). Similarly, there are also studies on the prognosis of PLR in CRC, but the research results are inconsistent or even contradictory; the PLR cutoff values used in these studies are also inconsistent. The prognostic effect of PLR on mCRC has not been systematically studied; therefore, this study was conducted to better reveal the prognostic value of PLR for mCRC.

## 2. Materials and Methods

### 2.1. Literature Search Strategy

The PubMed, Web of Science, and Embase (via OVID) were systematically searched to obtain all relevant research (up to March 2021). Search strategies included “PLR,” “platelet-lymphocyte ratio,” “platelet-to-lymphocyte ratio,” “platelet to lymphocyte ratio,” “platelet-lymphocyte,” “CRC,” “colorectal cancer,” “colon cancer,” “rectal cancer,” “prognosis,” “survival,” and “outcome.” There were no language restrictions in our study. Two reviewers screened the title and summary of each study individually. Once the relevant study was confirmed, the full text of the further evaluation will be available. This is a meta-analysis study and does not require ethical approval.

### 2.2. Inclusion and Exclusion Criteria

The including criteria of this meta-analysis were as follows: (1) all patients were pathologically diagnosed as mCRC, (2) the PLR was obtained from peripheral blood test before treatment, (3) the associations between PLR and OS and progression free survival (PFS) were investigated, (4) HR and 95% CI could be obtained by multivariate Cox regression analysis.

The excluding criteria of this meta-analysis were as follows:(1) irrelevant research, animal experiment, cell experiment, literature review, comment, letter, meta-analysis, or case report; (2) patients having other primary tumors; (3) insufficient prognosis data to estimate HR and 95% CI; (4) failed to provide cutoff value; and (5) if the same author reported patient results in multiple publications, only the most relevant results would be selected.

### 2.3. Data Extraction

Two researchers independently collected the data. The following data were extracted: publication details (first author's surname, year of publication, and geographic area of study), demographic characteristics (number of patients, age, and gender), cancer and follow-up data (stage, treatment strategy, median/mean follow-up time, and survival analysis), PLR (assessment method and cutoff value), and cutoff value were used to determine the “high” PLR and the “low” PLR.

### 2.4. Quality Assessment

The Newcastle-Ottawa Quality Assessment Scale (NOS) was used to assess the quality of the included studies, which includes 3 criteria, namely, selection (0-4 points), comparability (0-2 points), and results (0-3 points). NOS score≧6 is defined as high quality.

### 2.5. Statistical Analysis

We used Cochrane's *Q* and *I*^2^ statistic to assess the heterogeneity of the included trials. Significant heterogeneity was defined as *P* < 0.1 and *I*^2^ > 50%, and then, a random-effects model was selected to pool the results of the study; *P* ≥ 0.1 and *I*^2^ ≤ 50% were considered to be a value indicating homogeneity, so a fixed-effects model was subsequently applied.

Publication bias was assessed using the Begg's test/Egger's test and funnel plot [19]. We used the “trim and fill” method to assess the impact of publication bias on the overall effect [20]. All calculations were performed using STATA 14.0. *P* < 0.05 was considered statistically significant.

## 3. Results

### 3.1. Description of the Included Studies

**Figure 1:**
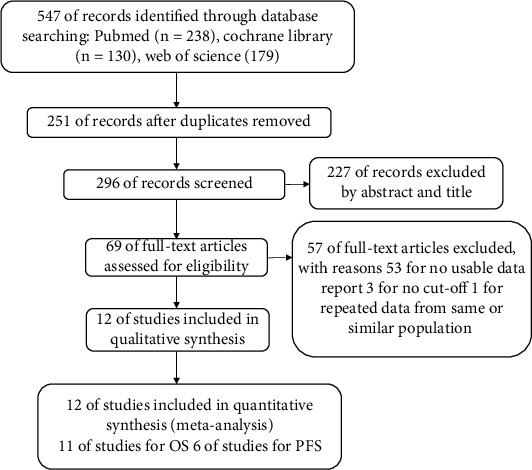
Flow chart of literature search and study selection.

The literature retrieval process is shown in Figure 1. According to the above search strategy, a total of 547 articles were finally identified. After removing the duplicates, 251 studies were excluded. By reading the titles and abstracts, 227 of the remaining 296 studies were further excluded. And then, 69 full-text articles have gone downloaded to evaluate their eligibility, in which 57 were excluded because noneffective data could be collected (*n* = 53), cutoff value was not recorded (*n* = 3), and repeated data from same or similar population (*n* = 1). Ultimately, 12 articles including 1452 patients published between 2014 and 2021 were included in this meta-analysis. The characteristics of the included studies are summarized in Table 1 [21–32]. All included literatures are retrospective studies. In the included articles, eleven investigated the prognostic role of PLR for OS and six for PFS. Eight of the included studies come from Eastern countries, including six from China, one from Turkey, and one from Japan. Four studies were from Western countries, one study from UK, one from Spain, one from Canada, and one from US. The PLR value was obtained by dividing the platelet count pretreatment by the lymphocyte count pretreatment.

### 3.2. Meta-Analysis Results

**Figure 2:**
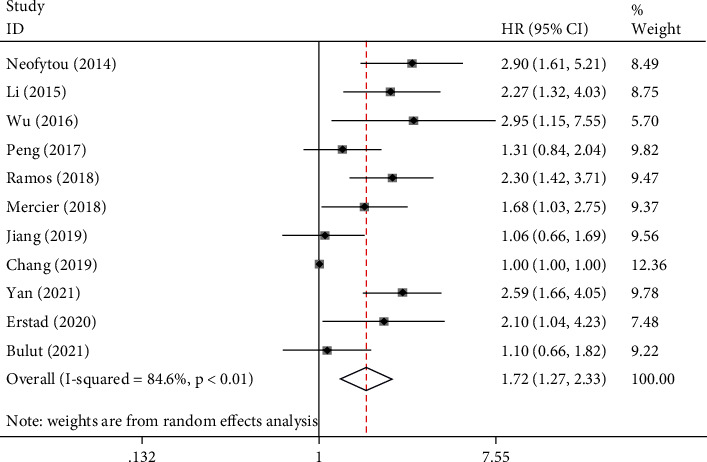
Results of prognostic analysis for PLR in mCRC for OS.

There were 11 studies to explore the prognostic significance of PLR for OS in patients with CRC. There was a significant heterogeneity between the studies (*I*^2^ = 84.6%, *P* < 0.01), so we chose the random-effects model to pool the results of the study. Pooled HR1.72 (95% CI: 1.27–2.33, *P* < 0.01, Figure 2) showed that patients with elevated PLR were expected to have lower OS after treatment.

**Figure 3:**
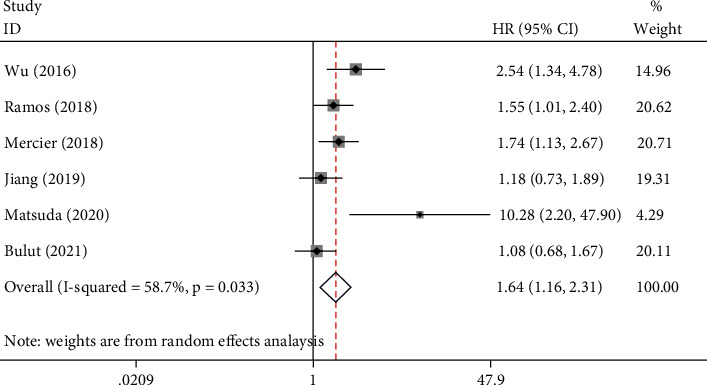
Results of prognostic analysis for PLR in mCRC for PFS.

There were 6 studies to explore the prognostic significance of PLR for PFS in patients with CRC. There was a significant heterogeneity between the studies (*I*^2^ = 58.7%, *P* = 0.033), so we chose the random-effects model to pool the results of the study. Pooled HR1.64 (95% CI: 1.16–2.31, *P* = 0.005, Figure 3) showed that patients with elevated PLR were expected to have lower PFS after treatment.

### 3.3. Publication Bias

**Figure 4:**
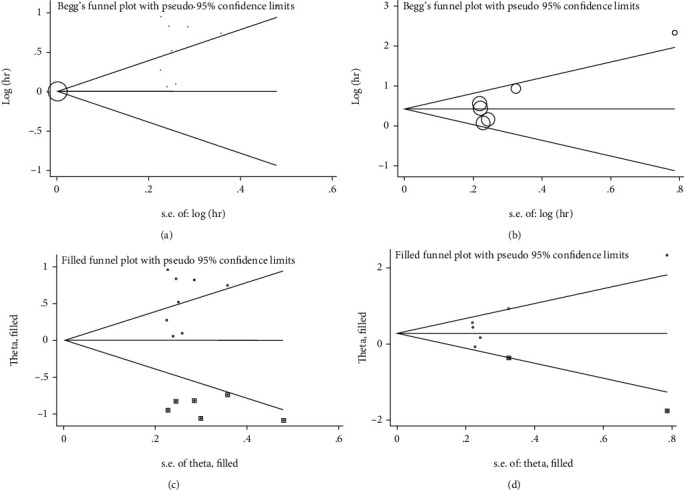
(a) Funnel plots assessing publication bias for OS. (b) Funnel plots assessing publication bias for PFS. (c) Trim-and-fill funnel plot for OS. (d) Trim-and-fill funnel plot for PFS.

Due to the heterogeneity in studies assessing the prognostic significance of PLR for OS and PFS, we used publication bias estimation to assess the reliability of meta-analysis results for these two indicators. We constructed funnel plots (Figures 4(a) and 4(b)), and the Egger's test indicated that publication bias was present, *P* = 0.001 and *P* = 0.056, respectively. Next, we used trim and fill methods to evaluate the symmetry of the funnel chart, by supplementing unpublished research, and the final result shows that there is no obvious asymmetry in the funnel chart obtained by adding 6 and 2 studies to the OS and PFS analysis, respectively, indicating no publication bias (Figures 4(c) and 4(d)).

### 3.4. Sensitivity Analyses

**Figure 5:**
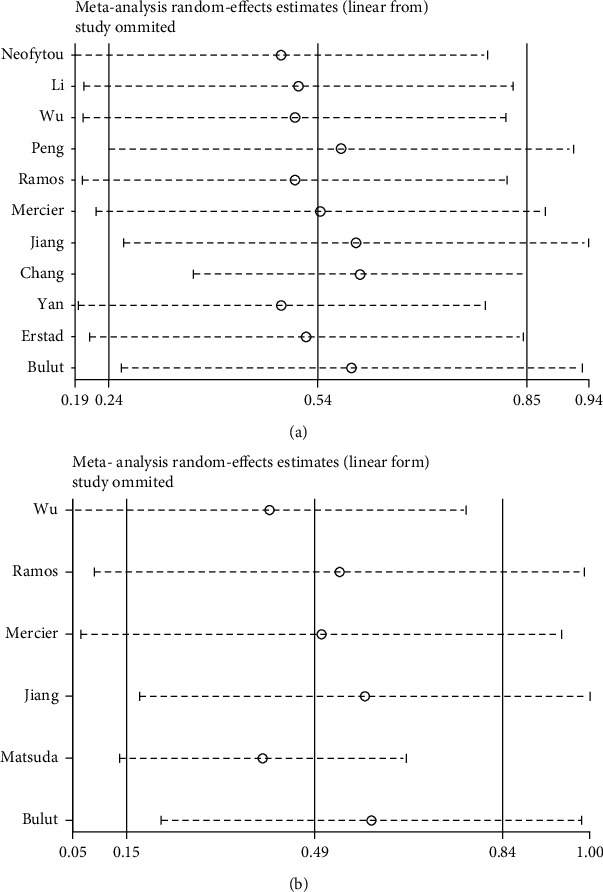
(a) Sensitivity analysis for the association between PLR and OS. (b) Sensitivity analysis for the association between PLR and PFS.

One study was deleted each time to reveal the influence of the individual data. When excluding any studies, the combined HR and its 95% CIs of OS and PFS were obviously unaffected, showing the stability of this analysis (Figures 5(a) and 5(b)).

### 3.5. Subgroup Analysis

We further explained the source of heterogeneity through subgroup analysis; we conducted this subgroup analysis based on the geographic region, sample size, PLR cutoff value, and major treatment therapy. Our results showed that high PLR predicted poor OS for all subgroups (Table 2).

## 4. Discussion

Recent studies have shown a correlation between PLR and the clinical outcome of mCRC, but there is conflicting evidence of the effect of PLR on the prognosis of patients with mCRC. This meta-analysis combined the results of 1452 patients with mCRC from 12 individual studies; we reassessed the prognostic role of PLR in mCRC. The results of this study indicate that patients with higher PLR levels before treatment have worse OS and PFS. We conducted a subgroup analysis to assess the prognostic significance of PLR, and the results showed that the prediction of PLR for OS is meaningful in all subsets.

Chronic inflammation plays an important role in different stages of tumor development; the underlying infections and inflammatory responses are associated with 15-20% of all deaths from cancer worldwide. Triggers of chronic inflammation that increase the risk of developing cancer are many, including microbial infections (for example, hepatitis virus and liver cancer), autoimmune diseases (for example, inflammatory bowel disease and CRC), and inflammatory conditions of unknown cause (for example, prostatitis and prostate cancer) [33]. Cancer-related inflammatory markers include inflammatory cells and inflammatory mediators present in tumor tissues; these inflammatory cells and mediators promote the occurrence and progression of cancer and participate in the migration, invasion, and metastasis of cancer cells. Among them, inflammatory markers such as platelets, lymphocytes, C-reactive protein, and Glasgow prognostic have been used in the study of tumor prognosis. Serum indicators have important prognostic value in mCRC; Silvestris et al. reported that low basal lactate dehydrogenases (LDH) levels and low pretreatment fibrinogen (FBG) serum levels are associated with favorable PFS and OS in mCRC patients; meanwhile, they found that medical treatment may influence LDH levels which showed a possible correlation between LDH changes and clinical outcome [34].

Platelets have been widely recognized as a component of the tumor microenvironment; the activation of platelets can release a variety of factors that regulate the tumor microenvironment, such as vascular endothelial growth factor (VEGF), TGF*β*1, fibroblast growth factor (FGF), and proinflammatory cytokines, which can affect tumor growth, tumor metastasis, tumor angiogenesis, tumor inflammation, and chemotherapy efficiency [35–37]. VEGF can induce the permeability of endothelial cells, promoting extravasation of cancer cells and promote angiogenesis at distant metastatic sites [38]. The synergistic effect of TGF*β*1 and platelet activating factor promotes tumor metastasis and induces the EMT process of tumor cell [37, 39]. Moreover, platelets easily interact with circulating tumor cells to induce EMT to promote metastasis [40–42]. Lymphocytes are closely related to tumor immunity; the immune tolerance of CD4+ T cells and the inhibition of CD8+ T cell activation can promote tumor immune escape and further promote tumor progression [43]. We divide the number of platelets by the number of lymphocytes to get the PLR value; an elevated PLR usually indicates an increase in the number of platelets or a decrease in the number of lymphocytes, which can lead to tumor progression and is associated with a poor prognosis.

There were some restrictions in our meta-analysis. First, the treatment method of each study was different, which may affect the relationship between PLR and OS or PFS. Second, the included studies were all retrospective studies, which may cause bias in the selection of patients. Third, because the sample size was small, including only six studies to assess the prognostic importance of PLR to PFS, which can be highly or underestimated, this also made us lack sufficient data to evaluate the association between PLR and disease free survival (DFS) and relapse free survival (RFS). Furthermore, due to the lack of appropriate data, the relationship between PLR and other important clinical parameters (such as age, sex, TNM staging, pathological type, tumor location, and neural invasion) has not been explored.

## 5. Conclusion

In conclusion, our meta-analysis showed that PLR is closely related with the survival outcome of mCRC patients. We can easily get the PLR value from routine blood tests, which is convenient for assessing the prognosis of mCRC patients and guiding individualized treatment. More studies are still needed to make up for the deficiencies of this analysis to improve the clinical utility of PLR.

## Figures and Tables

**Table 1:** Characteristics of studies included in the meta-analysis.

Study	Year	Country	Time	No.	TNM stage	Treatment	Follow-up (median)	Cutoff	Outcome	NOS
Neofytou	2014	UK	2005-2012	140	IV	None surgery	33 (1–103)	150	OS/DFS	8
Li	2015	China	2003-2012	110	IV	None surgery	NR	162	OS	7
Wu	2016	China	2008-2013	55	IV	None surgery	NR	150	OS/PFS	6
Peng	2017	China	2000-2012	150	IV	Surgery	36 (2–126)	150.17	OS/RFS	8
Ramos	2018	Spain	2003-2015	110	IV	None surgery	NR	172.4	OS/PFS	6
Mercier	2018	Canada	2008-2014	152	IV	None surgery	NR	330	OS/PFS	6
Jiang	2019	China	2010-2017	102	IV	None surgery	33.2 (2.6–94.5)	171.45	OS/PFS	7
Chang	2019	China	2012-2016	264	IV	None surgery	NR	173.48	OS	7
Matsuda	2020	Japan	2018-2019	21	IV	None surgery	13.1	193.2	OS/PFS	8
Yan	2020	China	1997-2013	103	IV	Surgery	55.4	144	OS	8
Erstad	2020	US	1995-2017	151	IV	Surgery	41.3 (2-186)	220	OS	8
Bulut	2021	Turkey	2010-2020	94	IV	None surgery	NR	180.36	OS/PFS	7

**Table 2:** Subgroup analysis for the association between elevated preoperative PLR and prognosis of patients with colorectal cancer.

	No.	HR (95% CI)	Log-rank *P* value	*I* ^2^%	*P* value
Geographic region					
Asia	7	1.492 (1.061-2.098)	0.021	81.4	<0.001
Non-Asia	4	2.164 (1.647-2.844)	0	0	0.563
Sample size					
<140	5	1.828 (1.271-2.629)	0.001	63.8	0.017
≥140	6	1.582 (1.042-2.403)	0.031	82.2	<0.001
Cutoff value					
<170	5	2.127 (1.673-2.703)	<0.001	42.6	0.138
≥170	6	1.383 (1.006-1.9)	0.003	75.2	<0.001
Treatment					
Surgery	3	1.901 (1.208-2.994)	0.006	57	0.097
None surgery	8	1.649 (1.16-2.343)	0.005	83.2	<0.001

## Data Availability

The data used in this meta-analysis can be obtained from the corresponding authors upon request.

## Abbreviations

CRC: Colorectal cancer
mCRC: Metastatic colorectal cancer
OS: Overall survival
PFS: Progression free survival
DFS: Disease free survival
RFS: Relapse free survival
PLR: Platelet to lymphocyte ratio
CI: Confidence interval
HR: Hazard ratio
NOS: Newcastle-Ottawa Scale.
